# Subclinical Myocardial Dysfunction Post Kawasaki Disease: A Systematic Review and Meta-Analysis

**DOI:** 10.3390/diseases14060188

**Published:** 2026-05-26

**Authors:** Dafni Charisopoulou, Sotiria Iliopoulou, Stelina Al Kagiet, Nikolaos Antoniou, Parthena Theodoridou, Kyriakos Tsantekidis, Eftsathios Oflidis, Christos Karakatsanis, Panagiotis Theodorou, George Koulaouzidis

**Affiliations:** 1 2nd Department of Paediatrics, AHEPA University General Hospital, School of Medicine, Aristotle University of Thessaloniki, 54636 Thessaloniki, Greece; dafnithess@yahoo.co.uk; 2 Cardiology Department, General Hospital G. Papanikolaou, 57010 Thessaloniki, Greece; sotiria.ili26@gmail.com (S.I.); salkayed@yahoo.gr (S.A.K.); nikosn771@gmail.com (N.A.); parth.k.theodoridou@gmail.com (P.T.); kktsante1@gmail.com (K.T.); oflidsta@gmail.com (E.O.); chriskar1705@gmail.com (C.K.); theod80@hotmail.com (P.T.); 3 Department of of Biochemical Sciences, Pomeranian Medical University, 70-204 Szczecin, Poland

**Keywords:** Kawasaki disease, global longitudinal strain, speckle-tracking echocardiography, myocardial dysfunction, meta-analysis

## Abstract

Background: Kawasaki disease (KD) is an acute childhood vasculitis with well-recognized coronary involvement, but subtle long-term myocardial dysfunction may persist despite preserved conventional systolic indices. Two-dimensional speckle-tracking echocardiography enables sensitive assessment of left ventricular global longitudinal strain (GLS). Methods: A systematic review and meta-analysis were conducted in accordance with PRISMA 2020. PubMed, Scopus, Web of Science, and Google Scholar were searched for studies evaluating post-acute GLS in children or adolescents with prior KD compared with healthy controls. The outcome measure was the mean difference (MD) in GLS (KD minus control). Random-effects models were used for the primary analysis. Results: Four case–control studies involving 192 patients with prior KD and 138 healthy controls were included in the quantitative synthesis. Compared with controls, patients with prior KD had significantly less negative GLS values, indicating worse longitudinal deformation (pooled MD, 0.77%; 95% CI, 0.18 to 1.36; Z = 2.34; *p* = 0.019). Between-study heterogeneity was low (Q = 3.17, I^2^ = 5.3%, tau^2^ = 0.030). Leave-one-out analysis showed that the overall direction of effect remained positive, although confidence intervals widened when individual studies were omitted. Interpretation of the funnel plot was limited by the small number of studies. Conclusions: Children and adolescents with a history of KD demonstrate a modest but significant reduction in LV GLS during follow-up, consistent with persistent subclinical myocardial dysfunction. Speckle-tracking echocardiography may provide incremental value in the long-term cardiac assessment of selected patients with prior KD.

## 1. Introduction

Kawasaki disease (KD) is an acute, self-limited systemic vasculitis of unknown etiology that predominantly affects children younger than 5 years of age and remains the leading cause of acquired heart disease in children in developed countries [1,2]. Its epidemiology demonstrates marked geographic variation, with incidence rates of approximately 10–20 per 100,000 children in Western countries and more than 300 per 100,000 in East Asian populations, particularly in Japan and Korea [3,4]. Clinically, KD is characterized by inflammation of medium-sized arteries, which primarily affects the coronary arteries. Untreated Kawasaki disease leads to coronary artery aneurysms in 15–25% of cases. Although early intravenous immunoglobulin therapy reduces this risk to below 5%, cardiovascular complications remain an important concern [1,2].

While coronary artery abnormalities are the most recognized complication of KD, myocardial involvement is also common during the acute phase. Transient myocarditis has been reported in up to 50–70% of patients and may manifest as reduced left ventricular systolic function, pericardial effusion, or valvular regurgitation [5,6]. Although these abnormalities typically resolve within weeks, evidence suggests that subtle myocardial injury may persist in the long term, even in patients without coronary artery aneurysms or in those considered fully recovered based on conventional imaging findings [7,8,9].

Conventional echocardiographic parameters, including left ventricular ejection fraction (LVEF) and fractional shortening, are frequently within normal ranges during follow-up and may lack sensitivity for detecting early or subclinical myocardial dysfunction [8,9,10]. In this context, two-dimensional speckle-tracking echocardiography (2D-STE) has emerged as a robust and angle-independent technique for assessing myocardial deformation. Global longitudinal strain (GLS), in particular, reflects the percentage change in myocardial length and is sensitive to dysfunction of subendocardial longitudinal fibers, which are often affected early in the disease process [11,12]. As such, GLS has gained increasing attention as an early marker of myocardial dysfunction across a range of cardiovascular conditions [13,14,15].

However, studies evaluating GLS in children with a history of Kawasaki disease have reported inconsistent findings [9,16,17,18,19]. These discrepancies may be related to differences in study populations, follow-up duration, coronary artery involvement, and methodological variability in strain measurement techniques [11,20]. As a result, the presence and extent of persistent subclinical myocardial dysfunction after KD remain uncertain.

Therefore, the aim of this systematic review and meta-analysis was to evaluate whether children and adolescents with a history of Kawasaki disease demonstrate persistent impairment in left ventricular global longitudinal strain compared with healthy controls during the post-acute phase (≥6 months after disease onset).

## 2. Materials and Methods

### 2.1. Study Design and Search Strategy

**Figure 1:**
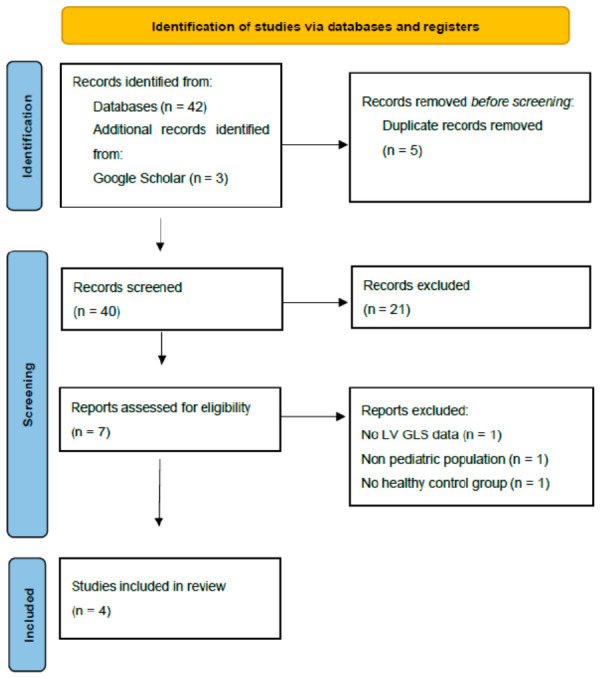
PRISMA 2020 flow diagram. The systematic search identified 45 records (42 from databases and 3 from Google Scholar). After the removal of 5 duplicates, 40 records underwent title and abstract screening. Nineteen records were excluded. Twenty-one full-text articles were sought for retrieval; however, fourteen reports could not be retrieved. Seven full-text articles were assessed for eligibility. Three studies were excluded after full-text review. Four (4) studies met the predefined inclusion criteria and were included in the quantitative synthesis.

This systematic review and meta-analysis were conducted in accordance with the Preferred Reporting Items for Systematic Reviews and Meta-Analyses (PRISMA) 2020 guidelines [21]. A predefined protocol was developed a priori to guide the review process; however, the protocol was not registered. The PRISMA checklist is provided as Supplementary Material (Table S1), and the study selection process is illustrated in the PRISMA flow diagram (Figure 1).

A comprehensive literature search was performed across PubMed, MEDLINE, Scopus, Web of Science, and Google Scholar in December 2025 without time restrictions. The search strategy combined Medical Subject Headings (MeSH) and free-text terms related to Kawasaki disease and myocardial deformation imaging. To improve sensitivity, no restrictions requiring explicit mention of “left ventricle” in the title or abstract were applied. Reference lists of included studies and relevant review articles were also manually screened to identify additional eligible studies.

Study selection was conducted in two stages. First, titles and abstracts were screened independently by two reviewers to identify potentially eligible studies. Second, full-text articles were assessed against predefined inclusion and exclusion criteria. Discrepancies between reviewers were resolved through discussion or consultation with a third reviewer.

Two reviewers independently screened titles and abstracts retrieved from PubMed, MEDLINE, Web of Science, and Google Scholar in December 2025. The final search strategy included the following terms: (“Kawasaki disease” OR “mucocutaneous lymph node syndrome”) AND (“global longitudinal strain” OR “GLS” OR “global longitudinal peak strain” OR “GLPS” OR “myocardial deformation” OR “speckle tracking” OR “velocity vector imaging”) AND (“echocardiography” OR “speckle-tracking echocardiography”) AND (“children” OR “pediatric” OR “adolescent”). In addition, the reference lists of all included studies and relevant review articles were manually screened to identify any additional eligible studies. No language restrictions were initially applied.

When required data were not reported in a format suitable for analysis, attempts were made to contact corresponding authors to obtain additional information. Studies were included only if sufficient data were available for quantitative synthesis.

### 2.2. Inclusion and Exclusion Criteria

Studies were considered eligible if they met the following criteria: (1) included children or adolescents aged ≤18 years with a confirmed history of Kawasaki disease diagnosed according to established criteria; (2) evaluated participants during the post-acute phase, defined as ≥6 months after fever onset; (3) assessed left ventricular global longitudinal strain (LV GLS) using two-dimensional speckle-tracking echocardiography (2D-STE); (4) included a healthy control group without known cardiac disease; and (5) reported GLS values as mean ± standard deviation or provided sufficient data for their calculation. Only case–control studies were considered eligible for quantitative synthesis.

Studies were excluded if they: (1) evaluated patients during the acute phase (<6 months after onset); (2) reported only regional or layer-specific strain parameters without overall LV GLS; (3) lacked a healthy control group; or (4) were case reports, review articles, editorials, conference abstracts, or animal studies. In addition, studies comparing Kawasaki disease with other inflammatory conditions, such as multisystem inflammatory syndrome in children (MIS-C), without inclusion of a healthy control group were excluded from the meta-analysis.

### 2.3. Data Extraction and Risk of Bias Assessment

Two authors independently extracted data from the included studies, and any discrepancies were resolved by a third reviewer. Extracted information included study characteristics (first author, year of publication, country, and study design), participant characteristics (sample size), follow-up duration, reported GLS values (mean ± SD) for KD and control groups. When required data were unavailable, attempts were made to contact the corresponding authors.

Methodological quality was assessed using the Newcastle–Ottawa Scale (NOS) for case–control studies, evaluating selection (up to 4 stars), comparability (up to 2 stars), and outcome/exposure (up to 3 stars) [22]. Studies were classified as having low, moderate, or high risk of bias according to prespecified thresholds. Studies scoring 7–9 were considered high quality.

### 2.4. Outcome Measure and Statistical Analysis

The primary outcome was the mean difference (MD) in LV GLS (%): MD = GLS_KD − GLS_Control. A positive MD indicates less negative (worse) strain in the KD group.

Statistical analysis was performed using Review Manager (RevMan) software (version 5.4 or later) and R with the metafor package. A random-effects model (DerSimonian–Laird method) was chosen a priori to account for expected clinical and methodological heterogeneity. Heterogeneity was quantified with Cochran’s Q, I^2^, and τ^2^. Sensitivity analysis included leave-one-out evaluation. Additional sensitivity analyses were performed using restricted maximum likelihood (REML) estimation and Hartung–Knapp/Sidik–Jonkman (HKSJ) confidence intervals to assess the robustness of the findings in the context of a small number of studies. Publication bias was assessed visually via funnel plot (limited by the small number of studies). A *p*-value < 0.05 was considered statistically significant.

## 3. Results

### 3.1. Study Selection

The systematic search retrieved 45 records, including 42 from electronic databases and 3 identified through Google Scholar. After removal of 5 duplicates, 40 records underwent title and abstract screening, of which seven studies were assessed for eligibility in full text [23,24,25,26,27,28,29]. Quantitative pooling was restricted to studies reporting left ventricular 2D-GLS as mean ± standard deviation in both KD and healthy control groups, allowing calculation of mean differences (MD). Studies using alternative comparator groups, reporting stage-specific or layer-specific strain analyses, or evaluating deformation parameters other than GLS were not eligible for meta-analysis. Three studies excluded after full-text review and the reasons for exclusion are summarized in Table 1, [27,28,29]. Four studies fulfilled all eligibility criteria and were included in the quantitative synthesis, Figure 1 [23,24,25,26]. A detailed list of studies excluded after full-text assessment, along with reasons for exclusion, is provided in Supplementary Table S2. When necessary, corresponding authors were contacted to obtain additional data required for inclusion in the meta-analysis.

### 3.2. Risk of Bias

Overall methodological quality was high. Two studies scored 9 of 9 and two scored 8 of 9 on the Newcastle–Ottawa Scale, indicating adequate case and control selection, satisfactory comparability, and appropriate ascertainment of outcomes. Detailed risk-of-bias assessments are presented in Table 2.

### 3.3. Study Characteristics

The four included studies were published between 2017 and 2024 and were conducted in Turkey (n = 2), China (n = 1), and Iran (n = 1). All studies employed a case–control design. The pooled sample included 192 patients with Kawasaki disease and 138 healthy controls. Mean participant age ranged from 3.6 to 12.0 years. Follow-up ranged from more than 6 months to more than 7 years after the acute illness. Coronary artery status varied; some studies included mixed populations (with/without CAA), while others focused on those without persistent aneurysms. All studies assessed LV GLS using 2D-STE. Detailed study characteristics are presented in Table 3.

Individual study findings:

- Dedeoglu et al. reported slightly lower GLS values in children with a history of Kawasaki disease compared with healthy controls (−23.10 ± 3.50% vs. −24.00 ± 3.10%) during mid-term follow-up, suggesting mild residual impairment in myocardial deformation [25].
- In contrast, Kayabey et al. found comparable GLS values between the two groups (−19.47 ± 3.56% vs. −19.08 ± 3.11%), supporting the possibility of functional normalization during longer-term follow-up [18].
- Lin et al. in a larger cohort with extended follow-up, demonstrated a more evident difference between patients with prior Kawasaki disease and controls (−20.71 ± 2.39% vs. −21.84 ± 2.18%), indicating persistent subclinical abnormalities in left ventricular mechanics [23].
- Similarly, Moghadam et al. reported subtle abnormalities in segmental strain parameters despite relatively preserved global values in some echocardiographic views, suggesting that regional myocardial dysfunction may persist even when overall GLS appears largely maintained [24].

### 3.4. Quantitative Synthesis of Global Longitudinal Strain

**Figure 2:**
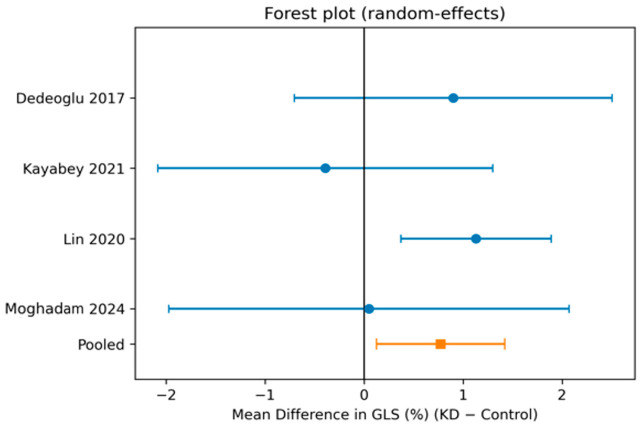
Forest plot showing the pooled mean difference (MD) in left ventricular global longitudinal strain (2D-GLS) between patients with Kawasaki disease and healthy controls. Positive values indicate less negative GLS and therefore worse longitudinal systolic deformation in Kawasaki disease. A random-effects model was applied [18,23,24,25].

Across individual studies, patients with prior KD generally demonstrated less negative GLS values than controls, consistent with impaired longitudinal myocardial deformation despite preserved conventional systolic function. Using a random-effects model, the pooled mean difference was 0.77% (95% CI 0.13 to 1.42; Z = 2.34; *p* = 0.019). A positivemean difference indicates less negative GLS in patients with prior KD and therefore worse systolic deformation (Figure 2).

### 3.5. Heterogeneity

Between-study heterogeneity was low (Table 4), with Cochran’s Q = 3.17, I^2^ = 5.5%, and τ^2^ = 0.031. The low I^2^ value indicates good consistency across studies and supports the robustness of the pooled estimate.

### 3.6. Sensitivity Analysis

**Figure 3:**
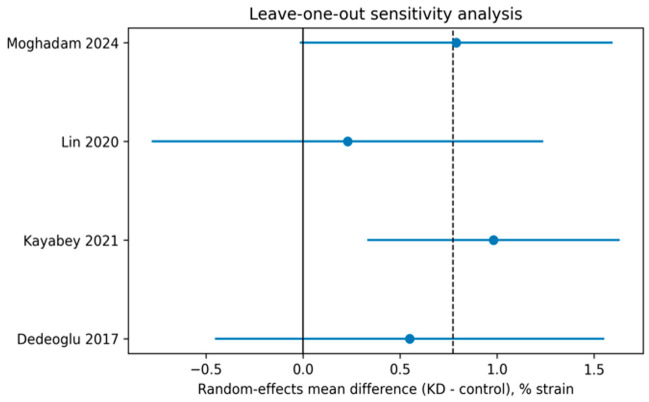
Leave-one-out sensitivity analysis demonstrating the pooled random-effects mean difference in 2D-GLS after sequential exclusion of individual studies. The overall effect estimate remained stable, indicating that no single study disproportionately influenced the pooled result [18,23,24,25].

Leave-one-out sensitivity analysis demonstrated that the pooled effect size remained stable after sequential exclusion of individual studies. No single study exerted a disproportionate influence on the overall estimate, Figure 3. Sensitivity analyses using REML estimation and HKSJ confidence intervals yielded results consistent with the primary analysis.

### 3.7. Publication Bias

**Figure 4:**
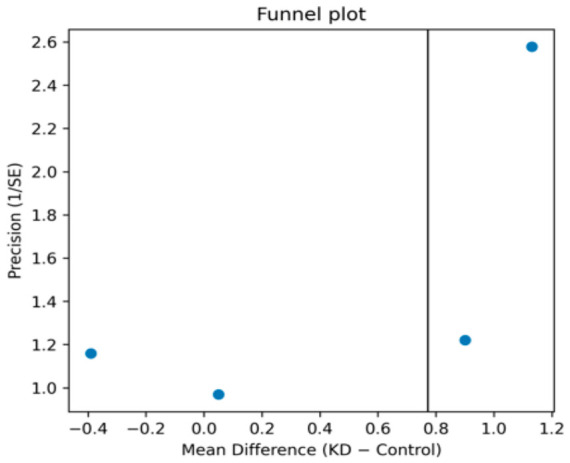
Funnel plot of standard error versus mean difference (MD) for studies included in the quantitative meta-analysis. Visual inspection did not suggest marked asymmetry; however, the small number of included studies limits the reliability of publication bias assessment.

Visual inspection of the funnel plot did not suggest substantial asymmetry, Figure 4. However, the small number of included studies limits the reliability of formal publication bias assessment. Formal assessment of small-study effects is limited due to the small number of included studies.

## 4. Discussion

### 4.1. Findings

In this systematic review and meta-analysis, we found that children and adolescents with a history of Kawasaki disease exhibit a small but statistically significant reduction in left ventricular global longitudinal strain compared with healthy controls during long-term follow-up. Although conventional systolic parameters such as ejection fraction are generally preserved, these findings suggest the presence of subtle abnormalities in myocardial deformation detectable by speckle-tracking echocardiography. While the magnitude of this difference is modest, it may reflect subclinical myocardial involvement, although its clinical significance remains to be established.

The included studies showed low between-study heterogeneity, and sensitivity analyses confirmed the robustness of the findings across different populations, imaging protocols, and follow-up intervals. These results support the presence of persistent subclinical myocardial alterations even years after apparent clinical recovery.

### 4.2. Pathophysiological and Imaging Considerations

The observation of reduced global longitudinal strain in patients with prior Kawasaki disease, despite preserved conventional systolic function, suggests that myocardial recovery following the acute inflammatory phase may be incomplete. During the acute phase of KD, myocardial inflammation is well documented and may involve both the interstitium and myocardial tissue more broadly. Even after resolution of overt inflammation, residual structural changes such as interstitial fibrosis or alterations in myocardial architecture may persist, potentially impairing myocardial deformation [30,31,32,33].

Microvascular and endothelial dysfunction may also contribute to persistent abnormalities in GLS. Subclinical myocardial injury after KD may reflect not only prior inflammatory involvement but also subtle alterations in myocardial perfusion and tissue integrity, even in the absence of overt epicardial coronary artery abnormalities. Given that subendocardial fibers are particularly vulnerable to ischemia due to their higher metabolic demand, these processes may preferentially affect longitudinal myocardial mechanics [30,31,32,33].

Another potential explanation relates to myocardial remodeling following inflammatory injury. Changes in myocardial fiber orientation, extracellular matrix composition, and ventricular compliance may alter myocardial mechanics in ways not captured by traditional volumetric indices such as LVEF. Speckle-tracking-derived parameters, including GLS, are more sensitive to these changes and may therefore detect early or subclinical abnormalities [34,35,36].

From an imaging perspective, GLS predominantly reflects subendocardial fiber function, which is particularly susceptible to ischemic and inflammatory injury. Accordingly, the modestly less negative GLS values observed in patients with prior KD may represent residual subendocardial dysfunction that is not detected by conventional echocardiographic parameters such as LVEF [34,35,36].

These findings support the concept that myocardial involvement in Kawasaki disease may persist beyond the acute phase and may be detectable only with sensitive myocardial deformation imaging techniques [5,37].

#### Comparison with the Previous Literature

Previous observational studies evaluating LV strain following Kawasaki disease have reported heterogeneous findings. Several investigations have demonstrated persistent reductions in global or regional myocardial strain during mid-term follow-up, despite preserved conventional systolic parameters such as LVEF, suggesting ongoing subclinical myocardial dysfunction [10,23,25]. In contrast, other studies have reported normalization of strain indices during convalescence or at longer-term follow-up, indicating potential recovery of myocardial mechanics over time [19,26].

These discrepancies are likely multifactorial. Differences in study design, patient selection, and clinical characteristics—including age at evaluation, time elapsed since acute KD, and degree of coronary artery involvement—may significantly influence myocardial deformation measurements. In addition, variability in echocardiographic acquisition protocols, vendor-specific software, and strain analysis techniques may contribute to inconsistencies in reported GLS values [34,36]. The lack of standardized reference ranges for pediatric populations further complicates direct comparison across studies [34].

The present meta-analysis provides a quantitative synthesis of the available evidence, suggesting that impairment in GLS, although modest in magnitude, is statistically significant and consistent across studies with low heterogeneity. These findings support the presence of subtle myocardial abnormalities that may persist beyond the acute phase of the disease, even in the absence of overt systolic dysfunction [10,18,23,25].

The relatively small reduction in GLS observed in this analysis should be interpreted within a broader clinical context. Evidence from both pediatric and adult populations indicates that even minor alterations in GLS may reflect early myocardial dysfunction and may precede detectable changes in conventional systolic indices [34,35,36,38,39,40]. These observations highlight the sensitivity of speckle-tracking-derived parameters in identifying subclinical ventricular abnormalities and support the potential value of GLS as an early marker of myocardial involvement.

### 4.3. Diagnostic and Clinical Implications

From a diagnostic imaging perspective, our findings suggest that conventional echocardiography alone may underestimate myocardial involvement after Kawasaki disease. Incorporation of two-dimensional speckle-tracking echocardiography may improve detection of subtle ventricular dysfunction, particularly in patients with preserved ejection fraction and no overt coronary abnormalities.

However, the clinical significance of the observed difference in GLS remains uncertain. The observed difference in GLS, although statistically significant, is small in magnitude, highlighting the need to distinguish statistical from clinical significance. At present, these findings should be interpreted as evidence of subclinical myocardial alteration rather than overt ventricular dysfunction, and it is not yet clear whether such changes translate into clinically meaningful outcomes.

Routine incorporation of GLS assessment into standard follow-up protocols cannot currently be universally recommended. However, it may have a role in selected patients, particularly those with coronary artery involvement, delayed treatment, or inconclusive conventional imaging findings, where it may provide incremental information and support more individualized follow-up strategies.

#### 4.3.1. Strengths

This study has several methodological strengths. It was conducted in accordance with PRISMA 2020 recommendations and employed a comprehensive multi-database search strategy with strict inclusion criteria. Standardized data extraction and methodological quality assessment helped reduce bias. In addition, the quantitative synthesis demonstrated low heterogeneity, and sensitivity analyses supported the robustness of the findings, strengthening the credibility of the results.

#### 4.3.2. Limitations

Several limitations should be acknowledged. First, the small number of included studies limits statistical power and precludes subgroup analyses based on key clinical variables such as coronary artery involvement, treatment timing, and disease severity. Assessment of small-study effects was limited by the small number of included studies.

Second, all included studies were observational case–control designs and are therefore susceptible to selection bias and residual confounding.

Third, variability in echocardiographic equipment, acquisition protocols, and strain analysis techniques may have influenced GLS measurements, highlighting the need for standardization. Vendor dependency remains an important limitation in strain imaging, as differences in software algorithms and post-processing techniques may affect GLS measurements and inter-study comparability. Fourth, differences in patient characteristics and follow-up duration may affect myocardial recovery, while the lack of individual patient-level data limited further analyses.

Finally, the clinical significance of modest GLS reductions remains uncertain, and longitudinal studies linking GLS abnormalities to clinical outcomes are required. Important sources of clinical heterogeneity, including coronary artery involvement, follow-up duration, age, and treatment timing, could not be formally explored due to the limited number of studies.

#### 4.3.3. Future Directions

Future prospective, multicenter longitudinal studies with standardized speckle-tracking protocols are needed to clarify the temporal evolution and prognostic significance of GLS abnormalities after Kawasaki disease. Long-term studies examining associations between strain parameters and clinical outcomes, including arrhythmias, exercise capacity, and ventricular function, will be essential to determine the clinical relevance of these findings. Integration with biomarkers and complementary imaging modalities may further improve understanding of the underlying mechanisms.

## 5. Conclusions

In conclusion, children and adolescents with a history of Kawasaki disease demonstrate a small but significant reduction in left ventricular global longitudinal strain during long-term follow-up. These findings suggest the presence of subclinical myocardial involvement despite preserved conventional systolic function. However, the overall certainty of evidence is low due to the limited number of studies, modest sample size, and potential clinical and methodological heterogeneity. Further studies are needed to clarify the clinical significance and long-term implications of these findings.

## Figures and Tables

**Table 1:** Studies not included in the quantitative meta-analysis and reasons for exclusion from pooling.

Study	Comparison	Outcome	Reason Excluded
Hu et al. [27], 2022	KD vs controls	GLS and layer-specific strain across disease stages	Stage-based and layer-specific analysis not directly comparable with pooled GLS mean difference
Sanchez et al. [28], 2019	KD with vs without CAA	Strain rate and segmental mechanics	Focused on strain rate rather than a pooled GLS mean difference
Piccinelli et al. [29], 2022	KD vs MIS-C	Segmental and global GLS	Comparator group was MIS-C rather than healthy controls

**Table 2:** Risk of Bias (Newcastle–Ottawa Scale).

Study	Selection	Comparability	Outcome	Total	Quality
Lin et al. [23]	★★★★	★★	★★★	9	High
Moghadam et al. [24]	★★★★	★★	★★	8	High
Dedeoglu et al. [25]	★★★★	★★	★★★	9	High
Kayabey et al. [18]	★★★★	★★	★★	8	High

**Table 3:** Characteristics and extracted data of studies included in the quantitative meta-analysis assessing left ventricular global longitudinal strain (2D-GLS) in patients with Kawasaki disease.

Study	Country	Study Design	KD Patients (n)	Controls (n)	Mean Age (Years)	Time Since KD	GLS in KD (Mean ± SD, %)	GLS in Controls (Mean ± SD, %)
Lin [23], 2020	China	Case–control	100	51	12.0 ± 3.4	>7 years	−20.71 ± 2.39	−21.84 ± 2.18
Moghadam [24], 2024	Iran	Case–control	27	27	5.6 ± 2.2	>6 months	−22.31 ± 4.98	−22.36 ± 1.98
Dedeoglu [25], 2017	Turkey	Case–control	35	30	9.1 ± 3.2	>1 year	−23.10 ± 3.50	−24.00 ± 3.10
Kayabey [18], 2021	Turkey	Case–control	30	30	3.6 ± 2.2	3.6 years (mean)	−19.47 ± 3.56	−19.08 ± 3.11

**Table 4:** Statistical heterogeneity and model diagnostics of the meta-analysis.

Parameter	Value
Cochran’s Q	3.17
Degrees of freedom	3
I^2^ (%)	5.5
τ^2^ (DerSimonian–Laird)	0.031
Fixed-effect pooled MD	0.80 (95% CI 0.20–1.41)
Random-effects pooled MD	0.77 (95% CI 0.18–1.36)

## Data Availability

This study is based on previously published data. All data analyzed during this study are included in the cited references. No new datasets were generated.

## Supplementary Materials

The following supporting information can be downloaded at: https://www.mdpi.com/article/10.3390/diseases14060188/s1, Table S1. PRISMA checklist [41]; Table S2. Studies excluded after full-text assessment and reasons for exclusion [7,9,16,17,19,26,42,43,44,45,46,47,48,49,50,51,52,53].

## Abbreviations

The following abbreviations are used in this manuscript:

CAA	Coronary artery aneurysm
CI	Confidence interval
GLS	Global longitudinal strain
I^2^	I-squared statistic (heterogeneity index)
KD	Kawasaki disease
LVEF	Left ventricular ejection fraction
LV	Left ventricle/Left ventricular
MD	Mean difference
MIS-C	Multisystem inflammatory syndrome in children
NOS	Newcastle–Ottawa Scale
PRISMA	Preferred Reporting Items for Systematic Reviews and Meta-Analyses
Q	Cochran’s Q statistic
SD	Standard deviation
STE	Speckle-tracking echocardiography
τ^2^ (tau^2^)	Between-study variance in random-effects model
2D-STE	Two-dimensional speckle-tracking echocardiography
Z	Z-statistic
*p*	*p*-value
